# Laparoscopic spleen-preserving No. 10 lymph node dissection for advanced proximal gastric cancer in left approach: a new operation procedure

**DOI:** 10.1186/1477-7819-10-241

**Published:** 2012-11-12

**Authors:** Wang Jia-Bin, Huang Chang-Ming, Zheng Chao-Hui, Li Ping, Xie Jian-Wei, Lin Jian-Xian

**Affiliations:** 1 Department of Gastric Surgery, Fujian Medical University Union Hospital, No.29 Xinquan Road, Fuzhou, 350001, Fujian Province, China

**Keywords:** Stomach neoplasms, Spleen-preservation, Laparoscopy, Lymph node dissection

## Abstract

**Background:**

To explore the feasibility of laparoscopic spleen-preserving No. 10 lymph node dissection in a left-sided approach for advanced proximal gastric cancer.

**Methods:**

The clinical data of 32 patients with advanced proximal gastric cancer who underwent laparoscopic spleen-preserving No. 10 lymph node dissection from June 2010 to December 2011 were analyzed.

**Results:**

Laparoscopic spleen-preserving No. 10 lymph node dissection using a left-sided approach was successfully performed for all patients without open conversion. The mean operation time was 206.4±54.3 minutes, mean intraoperative blood loss was 68.2±34.1 ml, mean number of No. 10 lymph nodes dissected was 2.8±2.1, mean number of positive No. 10 lymph nodes was 0.6±1.2, and the incidence of No. 10 lymph node metastasis was 11.6%. The mean postoperative hospital stay was 11.3±1.5 days. The postoperative morbidity rate was 9.4%, and there was no postoperative death. Splenic lobar vessels of all 32 patients were anatomically classified and divided into three types: 4 patients had a single lobar vessel, 22 had two lobar vessels and 6 had three lobar vessels.

**Conclusions:**

Laparoscopic spleen-preserving No. 10 lymph node dissection for advanced proximal gastric cancer using a left-sided approach is technically feasible. It simplifies the complicated surgical procedure of No. 10 lymph node dissection and leads to the popularization and promotion of this technique.

## Background

Many studies have reported that splenic hilar lymph nodes (No. 10 lymph nodes)metastasis in proximal gastric cancer is detected in 9.8% to 20.9% of cases [1,2]. When a surgeon undertakes total gastrectomy with D2 lymph node dissection for advanced proximal gastric cancer, he must dissect No. 10 lymph nodes [3]. In earlier years, the surgeon must undertake total gastrectomy with pancreaticosplenectomy in order to dissect the No. 10 and No. 11 lymph nodes [4]. However, because of the high rate of morbidity and mortality, this procedure had been usedfor gastric cancer which directly invaded the body and tail of the pancreas or spleen. At the same time, pancreas-preserving splenectomyusing No. 10 lymph node dissection had the same rate of postoperative survival and recurrence as pancreatosplenectomy, and had a lower rate of morbidity and mortality. It has gradually replaced pancreatosplenectomy in total gastrectomy with D2 lymph node dissection [5-8]. However, because of advances in surgical concepts, improvementsin the anatomical techniques and the progress of organ retention, spleen-preserving No. 10 lymph node dissection has been more and more widely supported. Many surgeons have suggested that it is safe and feasible and that it also has the same radical effect as pancreas-preserving splenectomy [9-11].

With the development of laparoscopic surgery, a number of authors have presented their experiences with laparoscopic surgery for gastric cancer, but most authors only reported on laparoscopy-assisted distal gastrectomy (LADG) with D2 lymph node dissection in distal gastric cancer [12-15]. No. 10 lymph node dissection is required in laparoscopy-assisted total gastrectomy (LATG) with D2 lymph node dissection;the vessels in the splenic hilum are especially intricate and complex, making the technique of lymph node dissection difficult. Surgeons who undertake this procedure must be equipped with exquisite surgical skills. There are only a few reports about the application of LATG with D2 lymph node dissection. Some authors reported laparoscopic No. 10 lymph node dissection using a medial approach [16,17], but that requires more complex surgical skills and more insertion of the trocars. The procedure has not been widely promoted.

In this study, we describe our experience of laparoscopic spleen-preserving No. 10 lymph node dissection for advanced proximal gastric cancer and investigate whether laparoscopic spleen-preserving No. 10 lymph node dissection using a left-sided approach is an innovative and feasible procedure which can simplify this complicated operation.

## Methods

### Patients

The study group consisted of 32 patients from the Department of Gastric Surgery, Affiliated Union Hospital of Fujian Medical University from June 2010 to December 2011. Laparoscopic spleen-preserving No. 10 lymph node dissection using a left-sided approach was successfully performed for all patients. In this study, the surgeon (HuangChangming) had performed more than 500 cases of laparoscopy-assisted gastrectomy(LAG) with D2 lymph node dissection in gastric cancer before starting to perform this procedure.

All subjects were preoperatively confirmed to have gastric cancer by analyses of endoscopic biopsy specimens. Preoperative imaging studies were routinely performed following endoscopic examination, computed tomography (CT) scanning, ultrasonography (US) of the abdomen and endoscopic US. Patients having T4 gastric cancer preoperatively according to the Japanese classification of gastric carcinoma (JCGC) [4] were excluded from this study. Patients with enlargement and integration of No. 10 lymph nodes were not considered candidates for surgery. Intraoperative diagnostic laparoscopy,which included a complete examination of the peritoneal cavity and liver, was also performed in all patients,. We explained the surgical procedure to the prospective patients, including its advantages and risks, and obtained informed consent before the procedure. Written informed consent was obtained from the patient for publication of this report and any accompanying images.

### Surgical technique

**Figure 1:**
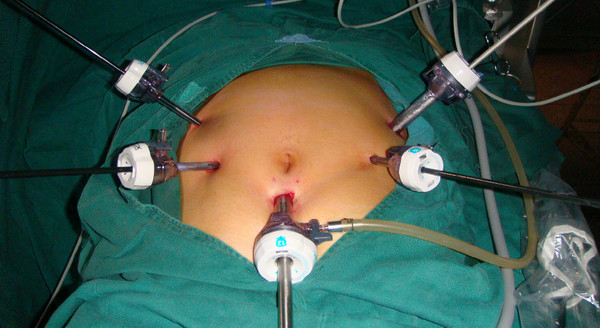
trocar site.

**Figure 2:**
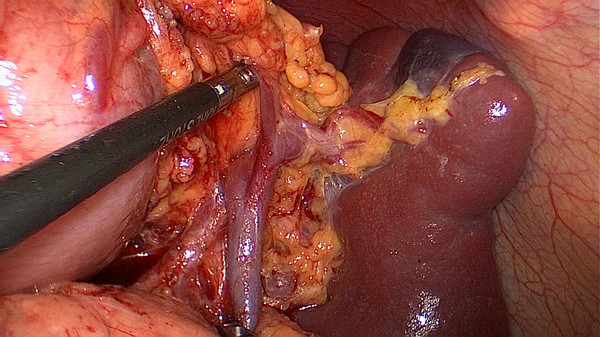
The left gastroepiploic vessels are vascularized at its origin.

The patient is placed in the reverse trendelenburg position with head elevated about 15 to 20 degrees, and tilted left side up about 20 to 30 degrees. One initial 10-mm trocar for a laparoscope is inserted below the umbilicus. Another 12-mm trocar is introduced in the left preaxillary line 2 cm below the costal margin as a major hand port. A 5-mm trocar is then inserted in the left midclavicular line 2 cm above the umbilicus as an accessory port, and a 5-mm trocar is placed at the contralateral site. A 5-mm trocar is inserted in the right preaxillary line 2 cm below the costal margin for exposure (Figure 1). The surgeon stands between the patient’s legs, the assistant and the camera operator are both on the patient’s right side. After dividing the left gastroepiploic vessels,the assistant pulls up the body of stomach toward the upper right and tenses the splenogastric ligament, the surgeon gently presses the tail of the pancreas toward the lower left, and the splenic hilum will become visible. The surgeon opens the pancreatic envelope, separates the membrane of the body and tail of the pancreas by ultrasonic scalpel to reach the posterior pancreas space at the superior border of the pancreas and reveals the end of the splenic vessels. The assistant pulls up the lymphatic fatty tissue on the surface of the lobe splenic vessels, then the surgeon opens the splenocolic ligaments near the end of the tail of the pancreas, the left gastroepiploic vessels are vascularized and clamped with its origin cut. The splenic lower pole vessels issue from the terminal branches of the splenic vessels before the root of the left gastroepiploic vessels in some patients. The surgeon should cut the left gastroepiploic artery above these vessels in order to avoid ischemia of the lower pole of the spleen (Figure 2).

**Figure 3:**
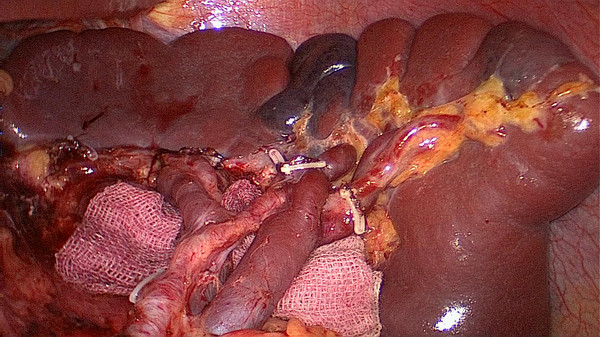
The fatty tissues including No. 10 lymph nodes are en-bloc removed from the splenic hilum (anterior).

**Figure 4:**
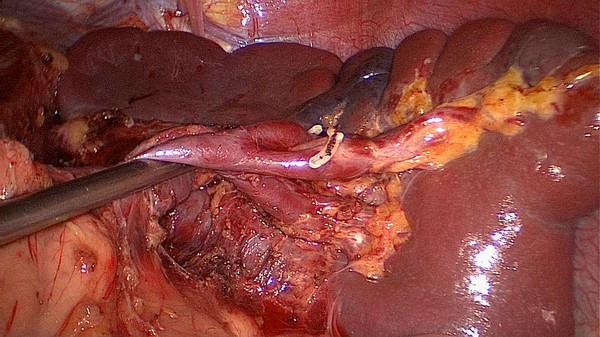
The fatty tissues including No. 10 lymph nodes are en-bloc removed from the splenic hilum (posterior).

**Figure 5:**
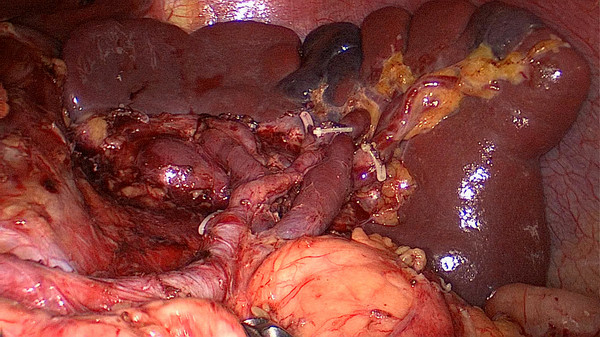
The fatty tissues including No. 11 lymph nodes are en-bloc removed from the splenic vessels.

No. 10 lymph node dissection: the assistant stretches the fundus and body of the stomach toward the upper right and keeps the tissue of the splenic hilum under tension. The greater omentumis then placed between the liver and the stomach. The surgeon presses the body and tail of the pancreas to expose the splenic hilum. At this time, the assistant gently pulls up the lymphatic fatty tissue at the surface of the branches of splenic vessels and keeps it under tension. The surgeon’s ultrasonic scalpel’s non-functional face closes the surface of the terminal branches of the splenic vessels. Starting from the root of the left gastroepiploic vessels, the surgeon carefully dissects the lymphatic fatty tissue in the splenic hilum from the lower left side to the upper right side. In the process of dissection, four to six branches of the short gastric vessels which issue from the splenic lobar vessels are skeletoned and divided at their roots. Cutting the last of the short gastric vessels should be appropriatelyfar away from the spleen. This can avoid a hemorrhage or ischemia of the spleen caused by injuring the upper pole of the splenic vessels (Figures 3, 4). After this procedure, the assistant puts the separation of the gastrosplenic ligaments between the liver and the stomach and continually pulls up the fundus and body of the stomach toward the upper right. The surgeon presses the pancreas and exposes the trunk of the splenic vessels which are located in the posterior pancreas space. The assistant pulls up the lymphatic fatty tissue which is separated from the surface of the splenic vessels. The surgeon dissects the lymphatic fatty tissue around the splenic vessels using an ultrasonic scalpel to separate the anatomic space from left to right. Then posterior gastric vessels are skeletoned and cut at the root where they issue from the trunk of the splenic vessels (Figure 5).

## Results

### Surgical outcome and postoperative course

Laparoscopic spleen-preserving No. 10 lymph node dissection using a left-sided approach was successfully performed for all patients without open conversion. None of the patients needed splenectomy because of vascular or spleen injury. Of the 32 patients, 26 were men and 6 were women. The mean age of the patients was 59.2±12.5 years. The mean operation time was 206.4±54.3 min, mean intraoperative blood loss was 68.2±34.1 ml, the mean number of No. 10 lymph nodes dissected was 2.8±2.1, the mean number of positive No. 10 lymph nodes was 0.6±1.2, and the incidence of No. 10 lymph node metastasis was 11.6%. The mean postoperative hospital stay was 11.3±1.5 days, the time to ambulation was 1.3±0.6 days, and the first flatus was 3.8±0.6 days. There was no postoperative deathbut the postoperative morbidity rate was 9.4%. There was one case with abdominal infection and two cases with pulmonary infection (Table 1).

**Table 1:** Patient characteristics and surgical outcomes

**Variable**	**Data**
Number	32
Age, years (mean±SD)	59.2±12.5
Sex	
male	26
female	6
Depth of invasion	
T2	12
T3	20
Lymph node metastasis	
N0	10
N1	10
N2	4
N3	8
BMI,kg/m^2^ (mean±SD)	22.1±3.5
Operation time, minutes (mean±SD)	206.4±54.3
Blood loss, ml (mean±SD)	68.2±34.1
Number of dissected lymph nodes, number (mean±SD)	35.4±9.7
Number of positive lymph nodes, number (mean±SD)	5.9±8.3
Number of dissected splenic lymph nodes, number (mean±SD)	2.8±2.1
Number of positive splenic lymph nodes, number (mean±SD)	0.6±1.2
Postoperative hospital stay, days (mean±SD)	11.3±1.5
The time to first flatus, days (mean±SD)	3.8±0.6
The time to ambulation, days (mean±SD)	1.3±0.6
Postoperative complications, number	3
abdominal infection,number	1
pulmonary infection,number	2
Postoperative morbidity rate,%	9.4

BMI, body mass index; SD, standard deviation.

### Anatomical classification of splenic lobar vessels

**Figure 6:**
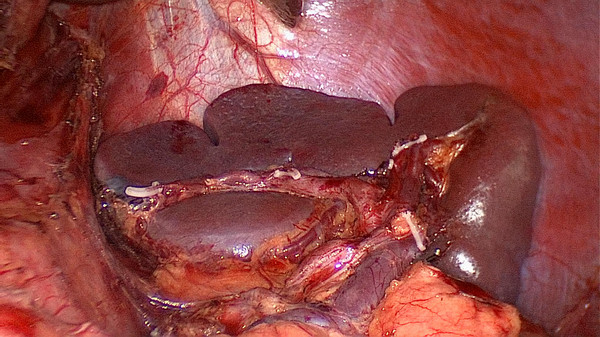
A single lobe splenic vessel.

**Figure 7:**
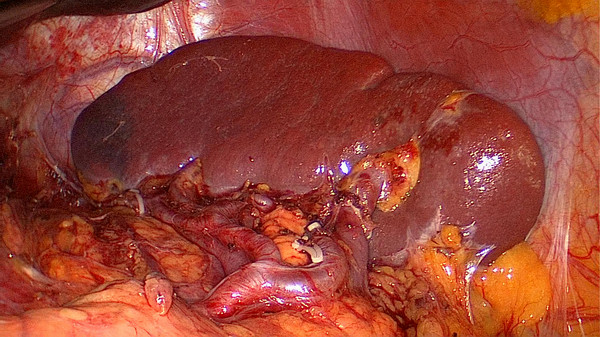
Two lobe splenic vessels.

**Figure 8:**
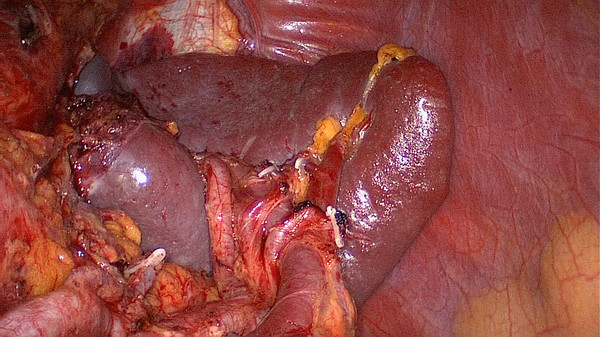
Three lobe splenic vessels.

Splenic vessels divide into the terminal branch of the splenic vessels (splenic lobar vessels) in the splenic hilum. We performed laparoscopic spleen-preserving No. 10 lymph node dissection using a left-sided approach for each patient. Splenic lobar vessels of all patients were anatomically classified and divided into three types [18]. Simultaneously, the splenic lobar artery was divided into three types: the splenic vessels in the splenic hilum that give rise to only one major superior lobar vessel alone into the spleen like an arc were called single branch type; the splenic vessels in the splenic hilum that give rise to two lobar vessels into the spleen (one superior and one inferior) were calledthe two branches type; and the splenic vessels in the splenic hilum that give rise to three lobar vessels into the spleen were called the three branches type(one superior, one middle and one inferior). The splenic lobar vessels of all patients were anatomically classified. In our study, the splenic lobar vessels had a single lobar vessel in 4 cases (Figure 6), two lobar vessels in 22 cases (Figure 7), and three lobar vessels in 6 cases (Figure 8).

## Discussion

Since Kitano *et al*. [19] first performed LADG for early gastric cancer in 1994, many studies have reported the benefits of laparoscopic gastric surgery. More and more patients who have gastric cancer receive laparoscopic surgery. Goh*et al*. [20] first performed LADG with D2 lymph node dissection for advanced gastric cancer in 1997, and produced good short-term results. Laparoscopic gastric surgery gradually expanded the indications for surgeryfrom early gastric cancer to moderately advanced gastric cancer. Therefore, more and more letters report about LADG with D2 lymph node dissection in patients with advanced lower gastric cancer. However, when the advanced proximal gastric cancer patients undergo laparoscopic D2 lymph node dissection, the No. 10 lymph node must be cleaned. In clinical practice, laparoscopic No. 10 lymph node dissection is considerably difficult and risky. A surgeon must skillfully master a difficult surgical technique in laparoscopic lymph node dissection to complete this operation. Therefore, only a few reports of LATG with D2 lymph node dissection were associated with advanced proximal gastric cancer.

Clinically, vessels in the splenic hilum are intricate and complicated;they are in a narrow space and this location is very deep. Both open and laparoscopic No. 10 lymph node dissection, dissection of these regional lymph nodes, is quite difficult. In open surgery, the spleen and distal pancreas must be mobilized from the retroperitoneum and No. 10 lymph nodes can be dissected thoroughly. However, this operation is traumatic and time-consuming. There is a risk of spleen walk or reverse after the operation. If the spleen and distal pancreas are not free, No. 10 lymph nodes are difficult to clean because of inadequate exposure. In laparoscopic surgery, we donot have the surgeon’s intuitive touch and exposure, and we only use laparoscopic grasping forceps for traction and separation in the local area. Therefore, we cannot intuitively judge the vessels’ shape, and it is easy to cause bleeding because of vascular injury. If surgeons donot have skilled laparoscopic operation experience, there would be increased bleeding and conversion from laparoscopy to laparotomy. Therefore, No. 10 lymph node dissection is a very important and difficult aspect of the treatment of patients with advanced proximal gastric cancer.

**Figure 9:**
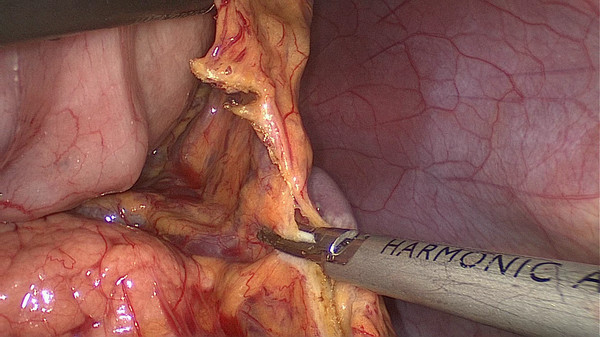
The stomach is pulled up toward the upper right,the splenogastric ligament will be in tension, and the splenic hilum and pancreatic tail will be visible.

**Figure 10:**
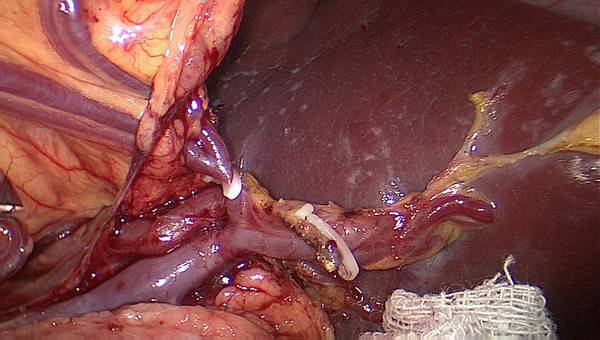
The short gastric vessels are freed, clamped, and cut at their origin.

**Figure 11:**
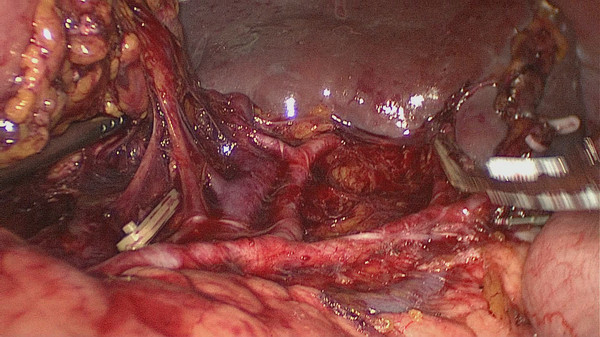
The splenic lobe vessels at the splenic hilum are exposed like a‘book’.

**Figure 12:**
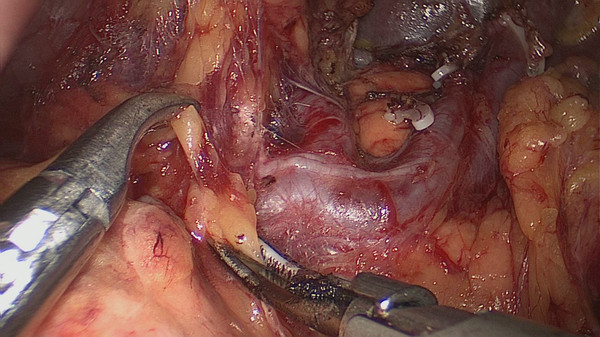
Dissect the No.11 lymph nodes using an ultrasonic scalpel separated along the anatomic space from the root of the splenic lobe vessels to the root to the splenic vessels.

Hyung *et al*. [16] first reported the application of laparoscopic spleen-preserving No. 10 lymph node dissection to the radical gastrectomy of proximal gastric cancer. The results showed that the average number of retrieved No. 10 lymph nodes was 2.7(1 to 5) in a group of 15 cases of upper gastric cancer; the rate of postoperative complication was 13.3%. Okabe *et al*. [17] also reported that seven patients with proximal gastric cancer underwent laparoscopic spleen-preserving No. 10 lymph node dissection. The average number of retrieved No. 10 lymph nodes was 2.6±2.8. They underwent No. 10 lymph node dissection using a medial approach. The surgeon stood on the patient’s right and punctured a more trocar below the xiphoid. No. 11p, 11d and 10 lymph nodes were dissected toward the distal splenic vessels from the root using ultrasonic shears. This procedure requires that the whole stomach is first extracted, to give a better exposure. One more insertion of the trocars for the lymph node dissection in the splenic hilum is necessary. We believe the fluency of this whole surgical procedure is disrupted and the concept of en-bloc resection for the lymph nodes with gastric cancer specimens is affected. Therefore, we developed a surgical procedure using a different approach, which is called laparoscopic spleen-preserving No. 10 lymph node dissection for advanced proximal gastric cancer using a left-sided approach, in order to achieve the effect of en-bloc resection. First, the surgeon operates between the patient’s legs, a camera operator is on the patient’s right side just beside the left side of the operator, and an assistant surgeon is on the patient’s right side. Separation of the greater omentum from the transverse colon is started from the left and continues rightward to the hepatic flexure, and the greater omentumis then placed between the liver and the stomach. The most important technique in our procedurethat is different from others is pulling up the stomach by the assistant without cutting the distal stomach. When the stomach is pulled up toward the upper right, the splenogastric ligament will be in tension, and the splenic hilum and pancreatic tail will be visible. In this procedure, the assistant can use a left hand grasping forceps to expose the splenic hilum in the operation field. We use the free stomach to ward off the organization influencing the operation visual field and keep the tissue of the splenic hilum in tension, the manual exposure of which is different from the practice of other surgeons. This is one of the most important aspects of our procedure. It is also important to get to the next step in the procedure in the No. 10 lymph node dissection (Figure 9). Secondly, exposing the root of the left gastroepiploic vessels is the key to the start of the No. 10 lymph node dissection. The surgeon presses the tail of the pancreas, pancreatic membrane is separated at the upper edge of the tail of the pancreas and the root of the left gastroepiploic vessels is exposed. At this point, the left gastroepiploic vessels are vascularized, clamped with its origin cut. The splenic lobar vessels in the inferior pole of the splenic hilum are exposed gradually. Thirdly, the surgeon dissects the No. 10 lymph nodes along the splenic lobe vessels from the inferior pole of the splenic hilum to the superior pole. In this procedure, the short gastric vessels are freed, clamped, and cut at their origin (Figure 10). When the assistant tensions the splenogastric ligament, with the splenic lobe vessels gradually exposed and the short gastric vessels cut, the lymph nodes around the splenic lobe vessels and distal splenic vessels are cleaned up. The splenic lobe vessels at the splenic hilum are exposed like a‘book’ from the lower left side to the upper right side and displayed in front of us. The fatty tissues including No. 10 lymph nodes are en-bloc removed from the splenic hilum with gastric cancer (Figure 11). Fourthly, the surgeon dissects the No. 11 lymph nodes using an ultrasonic scalpel, separated along the anatomic space from the root of the splenic lobe vessels to the root to the splenic vessels, which is from the left to the right side. Then the posterior gastric vessels are skeletoned and cut at the root which issues from the trunk of the splenic vessels (Figure 12). This is what we call the surgical procedure using a left-sided approach. It is difficult to perform this procedure using a medial approach. In our study, laparoscopic spleen-preserving No. 10 lymph node dissection was successfully performed for all patients without open conversion. The mean age of the patients was 59.2±12.5 years. The mean operation time was 206.4±54.3 min, the mean intraoperative blood loss was 68.2±34.1 ml, the mean number of No. 10 lymph nodes dissected was 2.8±2.1. The mean postoperative hospital stay was 11.3±1.5 days, the time to ambulation was 1.3±0.6 days and first flatus was 3.8±0.6 days. Splenic lobar vessels of all patients were anatomically classified and divided into three types.

## Conclusion

In conclusion, we believe laparoscopic spleen-preserving No. 10 lymph node dissection for proximal advanced gastric cancer using a medial approach is technically feasible. It simplifies the complicated surgical procedure of No. 10 lymph node dissection and leads to greater popularization and promotion.

## Competing interests

The authors declare they have no competing interests.

## Authors’ contributions

WJB and HCM conceived of the study, analyzed the data, and drafted the manuscript; ZCH helped revise the manuscript critically for important intellectual content; LP, XJW and LJX helped collect data and design the study. All authors read and approved the final manuscript.
